# Expression of laminin-1 and matrix metalloproteinase-9 in benign and malignant endometrium

**DOI:** 10.55730/1300-0144.5568

**Published:** 2022-11-30

**Authors:** Zehra KÜÇÜKAYDIN, Mustafa BAŞARAN, Yaşar ÜNLÜ, Ahmet BAŞARAN, Mertihan KURDOĞLU

**Affiliations:** 1 Department of Obstetrics and Gynecology, Private Konya Anıt Hospital, Konya, Turkey; 2 Başaran Clinic, Konya, Turkey; 3 Department of Pathology, Konya Training and Research Hospital, Konya, Turkey; 4 Department of Obstetrics and Gynecology, Faculty of Medicine, Kırıkkale University, Kırıkkale, Turkey

**Keywords:** Endometrium, endometrial neoplasms, endometrial hyperplasia, immunohistochemistry, laminin, matrix metalloproteinase 9

## Abstract

**Background/aim:**

Laminin-1 and matrix metalloproteinase (MMP)-9 may play roles in the progression from benign to malignant endometrium, so we aimed to investigate their levels of expression in these tissues.

**Materials and methods:**

This case-control study was conducted at a tertiary care center between January 2014 and December 2016. Paraffin blocks of 50 specimens of benign endometrium with proliferative (n = 20), secretory (n = 11), and atrophic (n = 5) endometrium; simple endometrial hyperplasia without atypia (n = 12); and endometrial polyp (n = 2) histology and 49 specimens of malignant endometrium with endometrioid (n = 40), serous (n = 7), clear cell (n = 1), and undifferentiated (n = 1) types were immunostained with laminin-1 and MMP-9 antibodies and assessed for basement membrane continuity for laminin-1 and the percentage and intensity of MMP-9 expression in epithelial cytoplasm.

**Results:**

Laminin-1 continuity in the basement membrane was higher in benign (92%) compared to malignant (16.3%) endometrium (p < 0.0001) without any difference between the subgroups within each group (p > 0.05). All atrophic endometria and endometrial polyps and 23.5% of low grade endometrioid and none of the other endometrial cancers showed uninterrupted basement membrane staining with laminin-1. All cases in malignant endometrium expressed MMP-9 with either low or high immunoreactivity while none of the cases in benign endometrium showed a high staining with MMP-9 (p < 0.01). Proliferative and hyperplastic endometrium together with grade 1 endometrioid cancer expressed MMP-9 better than the atrophic endometrium (p < 0.05). The immunoreactivity with MMP-9 increased gradually from secretory to hyperplastic endometrium and serous carcinoma (p < 0.05). MMP-9 expression in all types of cancers except grade 1 endometrioid and clear cell compared to proliferative endometrium was significantly higher (p < 0.05) and increased from proliferative to grade 2 endometrioid, grade 3 endometrioid, serous and undifferentiated endometrial carcinoma.

**Conclusion:**

Gradual increments in MMP-9 expression and basement membrane laminin-1 discontinuity may indicate progression from normal to hyperplastic and to low- and high-grade cancerous endometrium.

## 1. Introduction

In order to be able to achieve host tissue invasion and metastasize distantly, endometrial cancer cells have to attach to basement membrane laminin, leading to production of proteolytic enzymes degrading the extracellular matrix and migration across the basement membrane [1]. Laminin, the main noncollagenous glycoprotein and major constituent of all basement membranes with collagen IV, exists in multiple isoforms, among which laminin-1 (*α*1*β*1*γ*1) appears to be almost limitedly expressed in epithelial basement membranes [2]. In tumor invasion and metastasis, the key event, interaction of cancerous cells with laminin, is moderated by nonintegrin and integrin receptors of laminin, the expressions of which are altered in cancer [3]. Laminin receptor 1, which is a nonintegrin-type 67 kDa receptor of laminins (most studies are on laminin-1), is highly involved in tumor cell dissemination induced by laminin [3].

When compared to laminin-1-non-adherent cells, the cells adherent to laminin-1 were associated with reduction in apoptosis and increased activity in proliferation [4]. In addition, haptotactic, chemotactic, and migratory roles of laminin for tumor cells have also been reported [5,6]. Moreover, tumor cell invasion is also promoted by laminin through induction of proteases, which degrade various extracellular matrix components [7,8]. Human cancer cell lines of cervix (SiHa) and breast (MCF-7) cultured on a surface coated with laminin-1 have been shown to express matrix metalloproteinase-9 (MMP-9) [9,10], a 92-kDa member of the MMP family, which are calcium- and zinc-dependent metalloproteinases implicated in degradation of the extracellular matrix in normal as well as pathological conditions [11]. MMP-9 expression has been found to be modified under normal physiological states of the uterus as well as in uterine pathologies like infertility, myoma uteri, endometriosis, and breakthrough or dysfunctional bleeding [12–21]. MMP-9 appears to play important roles in various cancer types, including endometrial cancer [22–26].

These observations indicate the need for future studies on the link between epithelial basement membrane integrity and tumor behavior in normal and malignant endometrial tissues using antibodies to basement membrane protein laminin-1 and its degrading enzyme, MMP-9.

The aim of the present study was to examine the patterns of laminin-1 and MMP-9 expression immunohistochemically in benign versus malignant endometrium, and to determine whether observable alterations might be of value in characterizing the conversion of benign endometrium to malignant endometrium.

## 2. Materials and methods

### 2.1. Clinical data collection and handling of tissues

This case-control study was conducted at University of Health Sciences, Konya Training and Research Hospital between January 2014 and December 2016. After approval was granted by Necmettin Erbakan University Faculty of Medicine Institutional Review Board (IRB) on 15.11.2013 with the approval number of 2013-517, a total of 99 samples with the histological diagnoses of normal endometrium or benign endometrial pathologies (n = 50) and endometrial adenocarcinomas (n = 49) were obtained from the department of pathology. We purposely excluded the cases with atypical endometrial hyperplasia to include cases having exclusively benign or malignant features. The calculation of sample sizes in the present study were based on the studies by Soini et al. [22] and Di Nezza et al. [23], in which sample sizes of 29 had been shown to be sufficient. The human normal endometrial tissues had been obtained by probe-guided curettage during routine diagnostic and therapeutic procedures performed in the Department of Obstetrics and Gynecology. The human endometrial cancer tissues had been collected during oncologic surgery at the Gynecological Oncology Clinic of this department between 2014 and 2016. No chemotherapy or radiotherapy had been given to these patients before the surgical procedures including initial peritoneal washings followed by total abdominal or laparoscopic hysterectomies and bilateral salpingo-oophorectomies, as well as pelvic-paraaortic dissections of lymph nodes and cytoreductions when necessary. In addition to the multiple paraffin blocks, the clinical data and pathologic reports of the patients were also available from archives of the Obstetrics and Gynecology and Pathology departments.

### 2.2. Histopathological evaluation

Paraffin-embedded tissues of ninety-nine patients were used to obtain histologic sections stained with hematoxylin and eosin, which were rereviewed by a single pathologist (Y.Ü.). Noyes’ criteria were used in histologic dating [27]. The 1994 World Health Organization classification system was used in the classification of hyperplasia [28]. Biopsy specimens of the endometrium from patients 24 to 73 years of age and expressing no hyperplasia with atypia, apparent inflammation, or malignancy were examined; twenty were proliferative, eleven were secretory, and five were atrophic (from menopausal women lacking hormonal stimulation) endometrium, and twelve were simple hyperplasia without atypia (all from unopposed estrogen stimulation), whereas two were endometrial polyps. The endometrial cancers from the women between 45 and 84 years of age were endometrioid (n = 20, n = 14, and n = 6 for grade 1, grade 2, and grade 3, respectively), serous (n = 7), clear cell (n = 1), and undifferentiated (n = 1) histological types.

The grading scheme of International Federation of Gynecology and Obstetrics (FIGO) was used while grading the endometrioid type of endometrial cancer [29]. Endometrioid grade 1 or 2 cancers were defined as “low grade” and endometrioid grade 3 or nonendometrioid ones (clear cell, serous, and undifferentiated) as “high grade”, as proposed by International Society of Gynecological Pathologists [30].

### 2.3. Laminin-1 and matrix metalloproteinase-9 immunolocalization

The biotin–streptavidin indirect triple method was used for the immunohistochemical examination. Blocks embedded in paraffin were cut into tissue sections of 3 μm in thickness and put onto positively charged adhesion slides. The slides were immunohistochemically stained with rabbit polyclonal antibody to laminin-1 (RB-082-A1; Thermo Scientific, Fremont, CA, USA) diluted 1:100 and rabbit polyclonal antibody to MMP-9 (RB-9234-P; Thermo Scientific, Fremont, CA, USA) diluted 1:50 using an automated Leica BOND-MAX immunohistochemical device (Leica Microsystems, Cambridge, UK). The staining process was conducted using BOND Polymer Refine Detection kits (Leica Microsystems, Cambridge, UK) in accordance with the manufacturer’s instructions. Paraffin sections from the specimens were firstly deparaffinized and then rehydrated in alcohol and water with BOND Wash Solution (Leica Microsystems, Cambridge, UK) according to standard protocols. Antigenic masking was eliminated with BOND Epitope Retrieval Solution (Leica Microsystems, Cambridge, UK). The endogenic peroxidase activity was inhibited using peroxide blocking agent (Leica Microsystems, Cambridge, UK). Primary MMP-9 and laminin-1 antibodies were diluted. After washing, the antibodies were diluted again. The color reaction was performed with 3,3′-diaminobenzidine tetrahydrochloride (DAB). After washing, visualization of the sections was conducted with DAB and Mayer’s hematoxylin stain was used for counterstaining. Finally, distilled washing was automatically performed in the device, and the stained preparations were closed with a Leica CV5030 automatic closing device (Leica Microsystems, Cambridge, UK).

### 2.4. Evaluation of the slides

The slides of fifty tissues from normal endometrium or benign lesions of the endometrium and forty-nine tissues from malignant endometrium were examined immunohistochemically for the expression of laminin-1 in the basement membrane and of MMP-9 expression in the cell cytoplasm. The slides were evaluated with a light microscope (model BX51TF, Olympus, Tokyo, Japan) under 400× magnification by a single author (Y.Ü.) blinded to the clinical data of the patients.

Laminin-1 expression was evaluated as to whether it was stained throughout the basement membranes in a linear, uninterrupted, and continuous pattern or the staining was interrupted, being incomplete and absent in areas or in total. Only cytoplasmic and/or stromal staining was regarded as nonstaining.

The staining with MMP-9 was evaluated with two semiquantitative arbitrary scales. The first scale was used to categorize the percentage of epithelial cells with positive cytoplasmic staining as 0% (0), 1% to 10% (I), 11% to 50% (II), or 51% to 100% (III). The second scale was used to categorize the intensity of this staining as nonstaining (−), weak (+), moderate (++), or strong staining (+++). The histoscore was determined by multiplying the two values. The histoscore values from 0 to 9 were grouped to denote negative (0), low (1–5), and high (6–9) expression as described in the study by Jiraskova et al. [31]. The properties of staining for benign endometrium were compared with those for cancerous lesions of the endometrium.

### 2.5. Statistical analysis

SPSS Statistical Software for Windows, version 20.0 (SPSS Inc., Chicago, Illinois, USA) was used for the statistical analyses. The immunohistochemical data were presented as frequencies and percentages for categorical variables and analyzed with the Fisher’s exact χ^2^ test. For comparison of continuous variables which were expressed as mean ± standard deviation (SD), the unpaired two tailed Student’s t-test or the Mann–Whitney U test (in the case of skewed data) was used. p-values below 0.05 were considered statistically significant.

## 3. Results

In this immunohistochemical study, laminin-1 and MMP-9 expression levels were evaluated in benign and malignant endometrium tissue samples by using rabbit polyclonal antibodies to laminin-1 and MMP-9 antigens, respectively. The mean ages of patients with a normal endometrium and with endometrial cancer were statistically different (46.7 ± 8.5 and 61.6 ± 8.3 years, respectively) (p < 0.0001).

**Figure 1:**
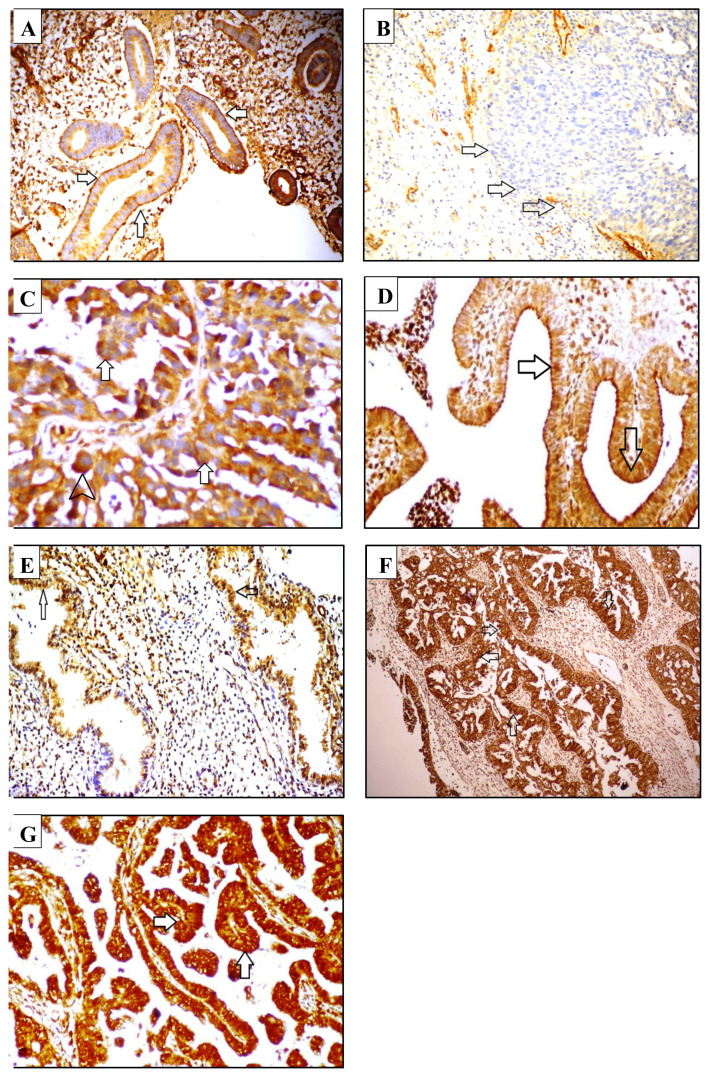
Examples of laminin-1 and matrix metalloproteinase (MMP)-9 immunohistochemical staining. **(A)** Proliferative endometrium showing continuous linear basement membrane laminin-1 immunopositivity (arrows). **(B)** Endometrioid-type endometrial carcinoma (grade 3) with basement membrane laminin-1 immunonegativity (arrows). **(C)** Serous-type endometrial carcinoma with cytoplasmic (arrowhead) and intercellular laminin-1 immunopositivity (arrows). **(D)** Simple endometrial hyperplasia without atypia showing moderate (++) cytoplasmic MMP-9 immunopositivity (arrows) in 11% to 50% (II) of cells. **(E)** Normal secretory endometrium with weak (+) cytoplasmic MMP-9 immunopositivity (arrows) in 11% to 50% (II) of cells. **(F)** Endometrioid-type endometrial carcinoma (grade 2) with moderate (++) cytoplasmic MMP-9 immunopositivity (arrows) in 11% to 50% (II) of cells. **(G)** Serous-type endometrial carcinoma showing strong (+++) cytoplasmic MMP-9 immunopositivity (arrows) in 51% to 100% (III) of cells. [original magnifications: 100× (A); 200× (B); 200× (C); 150× (D); 100× (E); 150× (F); 150× (G)].

In most of the benign endometrial tissues (92%) but in only 16.3% of the malignant endometrial lesions, linear and continuous staining of laminin-1 was evident in the basement membranes (p < 0.0001). Stained epithelium was surrounded by a narrow basement membrane band in the proliferative endometrium (Figure 1A). Distinct laminin positivity as a tortuous band was also evident in the endometrial glands of secretory endometrium stained with laminin-1. The basement membrane was distinctly visible in stained cases of simple endometrial hyperplasia without atypia. No significant difference was evident between subgroups within each group of benign and malignant endometria (p > 0.05). While the basement membranes of all atrophic endometria and endometrial polyps were stained with laminin-1 in a distinct linear, uninterrupted, and continuous manner beneath the epithelium, none of the high grade endometrioid (Figure 1B), serous, clear cell, or undifferentiated endometrial carcinomas expressed laminin-1 in their basement membranes. In 25% of grade 1 endometrioid endometrial carcinomas, a well-defined basement membrane was observed in the subepithelium. However, the basement membrane structure was incomplete in fifteen cases (75%) of grade 1 endometrioid endometrial carcinomas. In 21.4% of grade 2 endometrioid endometrial carcinomas, the areas of invasion were encircled by a continuous basement membrane, while no basement membrane was observed in invasion areas of 78.6% of grade 2 endometrioid endometrial carcinomas. Foci of intra- and intercellular staining for laminin were seen in one of the serous-type endometrial carcinomas (Figure 1C). The higher expression of laminin-1 in benign endometrium was observed in proliferative, secretory, and atrophic endometria together with simple endometrial hyperplasia without atypia against serous and all grades of endometrioid endometrial carcinomas (p < 0.05). However, the basement membranes of endometrial polypoid lesions expressed laminin-1 higher than only those of grade 3 endometrioid and serous carcinomas (p < 0.05). The results are detailed in Table 1.

In general, in all tissue types, a good correlation was observed between the percentages of cells stained positive with MMP-9 and the staining intensities of them evaluated with the two semiquantitative scales, as detailed. Based on histoscore on MMP-9 expression, all stained cases in benign endometrium showed a low immunoreactivity (78%), while the rate of nonstaining with MMP-9 (22%) was statistically higher than that for malignant endometrium (0%) (p < 0.01). In malignant endometrium, while 26.5% of slides expressed MMP-9 with a high immunoreactivity, none of the slides in benign endometrium showed a high staining with MMP-9 (p < 0.01). Benign and malignant endometrial lesions did not differ from each other with respect to low staining with MMP-9 (78% and 73.5%, respectively) (p > 0.05). The details are given in Table 2.

When compared to the proliferative endometrium, the rate of nonstaining with MMP-9 was higher (10% versus 80%, respectively) and the rate of low immunoreactivity with MMP-9 was lower (90% versus 20%, respectively) for the atrophic endometrium (p < 0.05) (Table 2). Low immunoreactivity was visualized in the cytoplasm of the only case of atrophic endometrium staining positive with MMP-9. All cases of simple endometrial hyperplasia without atypia showed low immunostaining with MMP-9 (Figure 1D), which was statistically higher than that for atrophic (20%) and secretory endometrium (63.6%) (p < 0.05). The results are detailed in Table 2.

Nonstaining with MMP-9 was highest in the atrophic endometrium (80%), which significantly differed from the serous and all grades of endometrioid endometrial carcinomas (0%) (p < 0.05). The secretory endometrium also showed a significantly higher percentage of nonstaining with MMP-9 (36.4%) compared to grade 1 and 2 endometrioid carcinomas (0%) (p < 0.05). MMP-9 expression in the stained cases of secretory endometrium was low (Figure 1E). A significantly higher percentage of slides expressed MMP-9 with a low immunoreactivity in grade 1 (90%) but not in grade 2 (71.4%) (Figure 1F) and grade 3 (66.7%) endometrioid carcinoma when compared with atrophic endometrium (20%) (p < 0.05). A significantly higher percentage of slides showed high immunoreactivity with MMP-9 in grade 2 (28.6%), grade 3 (33.3%), endometrial serous (57.1%), and undifferentiated endometrial carcinomas (100%) when compared to the proliferative endometrium (0%) (p < 0.05). Additionally, high immunoreactivity with MMP-9 was also significantly better in endometrial serous carcinoma when compared to the secretory endometrium and simple endometrial hyperplasia without atypia (0%) (p < 0.05) (Table 2). Between the subgroups of malignant endometrium, only endometrial serous carcinoma showed a significant difference from grade 1 endometrioid endometrial carcinoma with its higher immunoreactivity with MMP-9 (p < 0.05) (Figure 1G). The details are given in Table 2.

## 4. Discussion

Without any difference between the subgroups within each group, the basement membrane laminin-1 staining was mostly continuous in benign endometrium in contrast to malignant endometrium in which it was usually defective or discontinuous. While the cases in benign endometrium either did not stain or stained low with MMP-9, all cases in malignant endometrium expressed MMP-9 with either low or high immunoreactivity. A better expression of MMP-9 was observed in proliferative and hyperplastic endometrium together with grade 1 endometrioid cancer compared to the atrophic endometrium. The immunoreactivity with MMP-9 increased gradually from secretory to hyperplastic endometrium and serous carcinoma while its expression in all types of cancers except grade 1 endometrioid and clear cell compared to proliferative endometrium was significantly higher and increased from proliferative to grade 2, 3 endometrioid, serous, and undifferentiated endometrial carcinoma.

In functioning (proliferative, secretory), atrophic, and hyperplastic (simple endometrial hyperplasia without atypia, endometrial polyp) endometrium, a distinct basement membrane was visualized in the subepithelium and around the glands of the endometrium in nearly all tissues examined (46 out of 50) as narrow, continuous bands. In concordance with the results reported by Stenback et al. [32], Furness et al. [33], and Tanaka et al. [34], our observations with normal endometrium emphasize that the basement membranes are synthesized during the reproductive cycle of the endometrium, beginning from as early as the proliferative phase, and continues after the cessation of cell renewal and growth in the atrophic senile endometrium, which lacks constant hormonal stimulation. Although they were rarely seen in our tissues with normal epithelium (4 out of 50, all were either proliferative and secretory endometrium or simple endometrial hyperplasia without atypia), small defects and focal attenuation in laminin staining, which was also observed in the study by Vogel et al., have been associated with acute or chronic inflammation in these areas [35]. Similar to our findings, in the reports by Vogel et al. [35] and Bulletti et al. [36], endometrial hyperplasias had basement membranes that were generally intact but defective in some areas. A comparable result for endometrial polyps was also reported by Vogel et al. [35].

A distinct continuous basement membrane was evident only in almost a quarter (8 out of 34) of the low-grade endometrial cancers (endometrioid histological type) but in none of the high-grade cancers of the endometrium (grade 3 endometrioid, clear, serous, and undifferentiated). It has previously been reported that an intact basement membrane is rarely associated with carcinoma and having a basement membrane or not is the major discrepancy between the tumors with benign and malignant behavior [37]. Our demonstration of progressive loss of laminin-1 membranes with increasing degrees of anaplasia is similar to the findings reported by other workers [32,35,36] and it has been proposed to have resulted from either decreased production and deposition or increased degradation by the tumorous cells [32]. Bulletti et al. [36] linked this degradation with acquisition of enzymatic degradation activity based on previous studies [37,38]. The logic of progressive proliferation of endometrium from normal to hyperplastic and later to neoplastic state is based on a vicious cycle consisting of disappearance of the components of the basement membrane in glandular epithelial cells leading to increased permeability of the basement membrane to estrogen influx, endometrial proliferation and increased vasculature of the surface area, abnormal endometrial proliferation, resulting in hyperplasia and sometimes in endometrial adenocarcinoma, and in this way again the loss of basement membrane integrity [36].

It has been also commented that the finding of laminin containing basement membrane-like material in undifferentiated endometrial carcinoma tissues itself, which was also observed in one of our cases of serous carcinoma, might have been implicated by their limited ability left to produce basement membrane proteins [32]. Cytoplasmic accumulation of laminin in two cases of endometrioid carcinomas was interpreted as a sporadic abnormality in its synthesis and release by Vogel et al. [35].

In our study, MMP-9 was not expressed in most of the postmenopausal atrophic endometrium compared to the premenopausal proliferative endometrium. Similarly, in Laird et al.’s study, MMP-9 concentrations in premenopausal women were better than those in menopausal ones, which has been suggested as probably a reflection of atrophic endometrium associated with decreased secretory activity [39]. Nonchanging MMP-9 concentrations firstly in proliferative and later in secretory phases of the cycle were also a consistent finding between our study and the study by Laird et al. [39]. However, there are also some other reports suggesting that MMP-9 concentration diminishes during the early period of the secretory phase and subsequently increases late in the menstrual cycle with the beginning of menstruation [40,41]. One of the interesting results of our study was a significant increment in the expression of MMP-9 from nonfunctioning atrophic endometrium towards normal functioning proliferative endometrium, endometrium with hyperplasia (simple endometrial hyperplasia without atypia) or grade I endometrioid carcinoma. For MMP-9 expression, a significant gradual increment from proliferative endometrium towards grade 2 endometrioid, grade 3 endometrioid, serous and undifferentiated endometrial carcinoma together with a steady increase from secretory to hyperplastic endometrium and serous carcinoma were also evident. The findings of a previous study by Amalinei et al. [42] are consistent with ours.

In accordance with the findings of previous studies [24,39,42–44], our investigation also revealed that the expression of MMP-9 is upregulated in carcinomatous endometrium compared to benign endometrium. We noted that the expression of MMP-9 did not vary between the various histologic subtypes of endometrial cancer studied. This was just the opposite of the finding reported by Monaghan et al., whose study revealed significantly higher MMP-9 expression in endometrioid tumors versus serous ones [9]. Probably, with our too small serous endometrial cancer cases, a statistically significant difference for MMP-9 staining could not be reached compared to endometrioid type cancer (7 vs 40). However, similar to our findings, another study, by Graeslinn et al., did not reveal a difference in MMP-9 expression according to the histologic subtype [45]. Although increased production of MMP-9 has been linked to advanced grade in some reports [23,25,43,46], no such correlation was reported by Lopata et al. [24]. In our study, the histological grades of endometrioid endometrial carcinoma did not differ within themselves, but grade 1 endometrioid which is a low-grade cancer expressed MMP-9 less intensely than serous carcinoma which is a high-grade cancer.

To the best of our knowledge, this is the first immunohistochemical study investigating the possible roles of laminin-1 and MMP-9 together in transformation from the premalignant to malignant condition in the same pathological samples of benign, hyperplastic, and malignant endometrium. Uninterrupted, continuous expression of laminin-1 in the basement membranes of most of the benign endometrial tissues compared to discontinuous/defective or absent staining in basement membranes of the malignant endometrium was accompanied by increasing expression of MMP-9 from normal to hyperplastic endometrium and to cancerous lesions. This may be associated with the activation of an altered pathway of signal transduction by laminin-1 through a 67 kDa nonintegrin receptor of laminin or integrins on tumor cells, which results in basement membrane dissolution with collagenolysis by MMP-9 while normal tissue is becoming neoplastic [3]. In a study by Maity et al., a cell line of human cervical cancer (SiHa) cultured on a surface coated with laminin-1 induced the activation and expression of MMP-9 [47]. In another study, by Pal et al., a cell line of human breast carcinoma (MCF-7) cultured on a surface coated with laminin-1 also led to upregulation in MMP-9 expression together with diminished expression of tissue inhibitor of metalloproteinases-1 [10]. Both studies indicated that binding of these cell lines to laminin-1, probably by α2β1 integrin, induces signaling including focal adhesion kinase, phosphatidyl-inositol-3-kinase, extracellular signal regulated kinase, and nuclear factor-kappaB followed by upregulation of MMP-9 and cell migration [10,47]. Such a pathophysiological mechanism in tumor metastasis may also be valid for cancers of the endometrium and therefore similar investigations are warranted on them.

The basement membrane which separates the vascular endothelium and connective tissue from epithelium constitutes a barrier to the metastasis of tumor cells. For movement of tumor cells through the extracellular matrix to metastasize, its loss or remodeling is required. All components of extracellular matrix including the basement membrane can be degraded by active proteases including MMP-9 in vitro and they are frequently expressed at sites where the extracellular matrix is cleaved [48], as it was observed in our study. It has been shown that the staining of laminin–1 (also known as laminin–111) which is a large trimeric basement membrane glycoprotein is often discontinuous in breast tumors [49,50]. It was proposed that some cryptic sites that have biological activity may be exposed by the proteolytic cleavage of structural proteins. Four laminin-1-derived synthetic peptides that are active in malignancy have been described and three of them were shown to promote tumor growth by using different mechanism and cellular receptors [51].

This study has some limitations. The clinical-demographic data and endometrial biopsy indications of whole study population is lacking. Secondly, the small sizes of some histologic types of normal and malignant endometrium could have led to the observation of some statistically insignificant results. Thirdly, it would be interesting to evaluate endometrial hyperplasia with atypia and see whether laminin retention/loss and MMP expression are also changed in these tissues. It would be also great if we could evaluate the possible relation between the expression properties of laminin-1 and MMP-9 and the FIGO stages of forty-nine malignant cases together with their survival status through prognostic data.

## 5. Conclusion

Disease progression-related increase in MMP-9 and progressive loss of laminin-1 with increasing tumor grade may be involved in the progression from normal to hyperplastic and to low- and high-grade cancerous endometrium. On the molecular level, this transition may be implicated by the activation of cells by laminin-1 via altered signaling pathways, resulting in the induction of MMP-9-related basement membrane degradation, which needs to be clarified with further studies.

## Figures and Tables

**Table 1:** Immunostaining for laminin-1 in epithelial basement membranes of benign and malignant endometrium.

Type of tissue	Number of cases	Staining for laminin-1	
		Discontinuous/defective or absent	Continuous
Benign endometrium	50	4 (8)	46 (92)*
Proliferative	20	1 (5)	19 (95)†,‡,§,⁑
Secretory	11	2 (18.2)	9 (81.8)†,‡,§,⁑
Atrophic	5	0	5 (100)†,‡,§,⁑
Simple hyperplasia without atypia	12	1 (8.3)	11 (91.7)†,‡,§,⁑
Endometrial polyp	2	0	2 (100)§,⁑
Malignant endometrium	49	41 (83.7)	8 (16.3)
Grade 1 endometrioid	20	15 (75)	5 (25)
Grade 2 endometrioid	14	11 (78.6)	3 (21.4)
Grade 3 endometrioid	6	6 (100)	0
Endometrial serous	7	7 (100)	0
Endometrial clear cell	1	1 (100)	0
Undifferentiated endometrial	1	1 (100)	0

Results are expressed as the number (percentage)

* Significance when compared with the group of malignant endometrium, p < 0.0001

† Significance when compared with the group of grade 1 endometrioid endometrial carcinoma, p < 0.01

‡ Significance when compared with the group of grade 2 endometrioid endometrial carcinoma, p < 0.01

§ Significance when compared with the group of grade 3 endometrioid endometrial carcinoma, p < 0.05

⁑ Significance when compared with the group of endometrial serous carcinoma, p < 0.05

**Table 2:** Matrix metalloproteinase-9 expression in epithelial cytoplasm evaluated by histoscores (staining intensity × percentage of positive cells).

Type of tissue	Number of cases	Histoscore for MMP-9		
		Negative	Low	High
Benign endometrium	50	11 (22)*	39 (78)	0*
Proliferative	20	2 (10)†	18 (90)†	0
Secretory	11	4 (36.4)	7 (63.6)	0
Atrophic	5	4 (80)	1 (20)	0
Simple hyperplasia without atypia	12	0†,‡	12 (100)†,‡	0
Endometrial polyp	2	1 (50)	1 (50)	0
Malignant endometrium	49	0	36 (73.5)	13 (26.5)
Grade 1 endometrioid	20	0†,‡	18 (90)†	2 (10)
Grade 2 endometrioid	14	0†,‡	10 (71.4)	4 (28.6)⁑
Grade 3 endometrioid	6	0†	4 (66.7)	2 (33.3)⁑
Endometrial serous	7	0†	3 (42.9)⁑,§,⁂	4 (57.1)‡,⁑,§,⁂
Endometrial clear cell	1	0	1 (100)	0
Undifferentiated endometrial	1	0	0	1 (100)⁑

Results are expressed as the number (percentage)

The histoscore values from 0 to 9 were grouped to indicate negative (0), low (1–5) and high (6–9) expression

* Significance when compared with the group of malignant endometrium, p < 0.01

† Significance when compared with the group of atrophic endometrium, p < 0.05

‡ Significance when compared with the group of secretory endometrium, p < 0.05

⁂ Significance when compared with the group of proliferative endometrium, p < 0.05

§ Significance when compared with the group of simple endometrial hyperplasia without atypia, p < 0.01

⁑ Significance when compared with the group of grade 1 endometrioid endometrial carcinoma, p < 0.05

## References

[1] Turpeenniemi-Hujanen T Thorgeirsson UP Rao CN Liotta LA Laminin increases the release of type IV collagenase from malignant cells Journal of Biological Chemistry 1986 261 4 1883 1889 10.1016/S0021-9258(17)36025-8 3003087

[2] Ekblom P Lonai P Talts JF Expression and biological role of laminin-1 Matrix Biology 2003 22 1 35 47 10.1016/s0945-053x(03)00015-5 12714040

[3] Givant-Horwitz V Davidson B Reich R Laminin-induced signaling in tumor cells Cancer Letters 2005 223 1 1 10 10.1016/j.canlet.2004.08.030 15890231

[4] Kim WH Lee BL Kim DK Kleinman HK Laminin-1-adherent cancer cells show increased proliferation and decreased apoptosis in vivo Anticancer Research 1999 19 4B 3067 3071 10652594

[5] Aznavoorian S Stracke ML Krutzsch H Schiffmann E Liotta LA Signal transduction for chemotaxis and haptotaxis by matrix molecules in tumor cells Journal of Cell Biology 1990 110 4 1427 1438 10.1083/jcb.110.4.1427 2324200 PMC2116083

[6] Patarroyo M Tryggvason K Virtanen I Laminin isoforms in tumor invasion, angiogenesis and metastasis Seminars in Cancer Biology 2002 12 3 197 207 10.1016/S1044-579X(02)00023-8 12083850

[7] Engbring JA Kleinman HK The basement membrane matrix in malignancy Journal of Pathology 2003 200 4 465 470 10.1002/path.1396 12845613

[8] Khan KM Falcone DJ Role of laminin in matrix induction of macrophage urokinase-type plasminogen activator and 92-kDa metalloproteinase expression Journal of Biological Chemistry 1997 272 13 8270 8275 10.1074/jbc.272.13.8270 9079647

[9] Monaghan H MacWhinnie N Williams AR The role of matrix metalloproteinases-2, -7 and -9 and beta-catenin in high grade endometrial carcinoma Histopathology 2007 50 3 348 357 10.1111/j.1365-2559.2007.02612.x 17257130

[10] Pal S Moulik S Dutta A Chatterjee A Extracellular matrix protein laminin induces matrix metalloproteinase-9 in human breast cancer cell line mcf-7 Cancer Microenvironment 2014 7 1–2 71 78 10.1007/s12307-014-0146-6 24858419 PMC4150877

[11] Wang L Wang Q Li HL Han LY Expression of MiR200a, miR93, metastasis-related gene RECK and MMP2/MMP9 in human cervical carcinoma--relationship with prognosis Asian Pacific Journal of Cancer Prevention 2013 14 3 2113 2118 10.7314/apjcp.2013.14.3.2113 23679328

[12] Skinner JL Riley SC Gebbie AE Glasier AF Critchley HO Regulation of matrix metalloproteinase-9 in endometrium during the menstrual cycle and following administration of intrauterine levonorgestrel Human Reproduction 1999 14 3 793 799 10.1093/humrep/14.3.793 10221716

[13] Hickey M Higham J Sullivan M Miles L Fraser IS Endometrial bleeding in hormone replacement therapy users: preliminary findings regarding the role of matrix metalloproteinase 9 (MMP-9) and tissue inhibitors of MMPs Fertility and Sterility 2001 75 2 288 296 10.1016/s0015-0282(00)01690-3 11172829

[14] Vincent AJ Zhang J Ostor A Rogers PA Affandi B Decreased tissue inhibitor of metalloproteinase in the endometrium of women using depot medroxyprogesterone acetate: a role for altered endometrial matrix metalloproteinase/tissue inhibitor of metalloproteinase balance in the pathogenesis of abnormal uterine bleeding? Human Reproduction 2002 17 5 1189 1198 10.1093/humrep/17.5.1189 11980737

[15] Galant C Berliere M Dubois D Verougstraete JC Charles A Focal expression and final activity of matrix metalloproteinases may explain irregular dysfunctional endometrial bleeding The American Journal of Pathology 2004 165 1 83 94 10.1016/S0002-9440(10)63277-4 15215164 PMC1618526

[16] Chung HW Wen Y Chun SH Nezhat C Woo BH Matrix metalloproteinase-9 and tissue inhibitor of metalloproteinase-3 mRNA expression in ectopic and eutopic endometrium in women with endometriosis: a rationale for endometriotic invasiveness Fertility and Sterility 2001 75 1 152 159 10.1016/s0015-0282(00)01670-8 11163831

[17] Szamatowicz J Laudanski P Tomaszewska I Matrix metalloproteinase-9 and tissue inhibitor of matrix metalloproteinase-1: a possible role in the pathogenesis of endometriosis Human Reproduction 2002 17 2 284 288 10.1093/humrep/17.2.284 11821264

[18] Collette T Bellehumeur C Kats R Maheux R Mailloux J Evidence for an increased release of proteolytic activity by the eutopic endometrial tissue in women with endometriosis and for involvement of matrix metalloproteinase-9 Human Reproduction 2004 19 6 1257 1264 10.1093/humrep/deh290 15105396

[19] Collette T Maheux R Mailloux J Akoum A Increased expression of matrix metalloproteinase-9 in the eutopic endometrial tissue of women with endometriosis Human Reproduction 2006 21 12 3059 3067 10.1093/humrep/del297 16880228

[20] Inagaki N Stern C McBain J Lopata A Kornman L Analysis of intra-uterine cytokine concentration and matrix-metalloproteinase activity in women with recurrent failed embryo transfer Human Reproduction 2003 18 3 608 615 10.1093/humrep/deg139 12615834

[21] Inagaki N Ung L Otani T Wilkinson D Lopata A Uterine cavity matrix metalloproteinases and cytokines in patients with leiomyoma, adenomyosis or endometrial polyp European Journal of Obstetrics & Gynecology and Reproductive Biology 2003 111 2 197 203 10.1016/s0301-2115(03)00244-6 14597251

[22] Soini Y Alarakkola E Autio-Harmainen H Expression of messenger RNAs for metalloproteinases 2 and 9, type IV collagen, and laminin in nonneoplastic and neoplastic endometrium Human Pathology 1997 28 2 220 226 10.1016/s0046-8177(97)90110-6 9023406

[23] Di Nezza LA Misajon A Zhang J Jobling T Quinn MA Presence of active gelatinases in endometrial carcinoma and correlation of matrix metalloproteinase expression with increasing tumor grade and invasion Cancer 2002 94 5 1466 1475 10.1002/cncr.10355 11920503

[24] Lopata A Agresta F Quinn MA Smith C Ostor AG Detection of endometrial cancer by determination of matrix metalloproteinases in the uterine cavity Gynecologic Oncology 2003 90 2 318 324 10.1016/s0090-8258(03)00328-7 12893193

[25] Aglund K Rauvala M Puistola U Angstrom T Turpeenniemi-Hujanen T Gelatinases A and B (MMP-2 and MMP-9) in endometrial cancer-MMP-9 correlates to the grade and the stage Gynecologic Oncology 2004 94 3 699 704 10.1016/j.ygyno.2004.06.028 15350361

[26] Kamat AA Feng S Agoulnik IU Kheradmand F Bogatcheva NV The role of relaxin in endometrial cancer Cancer Biology & Therapy 2006 5 1 71 77 10.4161/cbt.5.1.2289 16322684

[27] Noyes RW Hertig AT Rock J Dating the endometrial biopsy Fertility and Sterility 1950 1 1 3 25 10.1016/s0015-0282(16)30062-0 31623748

[28] Silverberg SG Mutter GL Kurman RJ Kubik-Huch RA Nogales F Tumors of the uterine corpus: epithelial tumors and related lesions Tavassoli FA Devilee P World Health Organization Classification of Tumours Pathology and Genetics of Tumours of the Breast and Female Genital Organs Lyon, France IARC Press 2003 221 232

[29] Announcements International Federation of Gynecology and Obstetrics (FIGO) stages—1988 revision Gynecologic Oncology 1989 35 1 125 127

[30] Soslow RA Tornos C Park KJ Malpica A Matias-Guiu X Endometrial carcinoma diagnosis: Use of FIGO grading and genomic subcategories in clinical practice: Recommendations of the International Society of Gynecological Pathologists International Journal of Gynecological Pathology 2019 38 Suppl 1 (Iss 1 Suppl 1) S64 S74 10.1097/PGP.0000000000000518 30550484 PMC6295928

[31] Jiraskova L Ryska A Duintjer Tebbens EJ Hornychova H Cecka F Are ENT1/ENT1, NOTCH3, and miR-21 reliable prognostic biomarkers in patients with resected pancreatic adenocarcinoma treated with adjuvant gemcitabine monotherapy? Cancers (Basel) 2019 11 11 1621 10.3390/cancers11111621 31652721 PMC6893654

[32] Stenback F Risteli J Risteli L Wasenius VM Basement membrane laminin and type IV collagen in endometrial adenocarcinoma: relation to differentiation and treatment Oncology 1985 42 6 370 376 10.1159/000226066 3906481

[33] Furness PN Lam EW Patterns of basement membrane deposition in benign, premalignant, and malignant endometrium Journal of Clinical Pathology 1987 40 11 1320 1323 10.1136/jcp.40.11.1320 3693569 PMC1141232

[34] Tanaka T Wang C Umesaki N Remodeling of the human endometrial epithelium is regulated by laminin and type IV collagen International Journal of Molecular Medicine 2009 23 2 173 180 10.3892/ijmm_00000114 19148540

[35] Vogel HP Mendelsohn G Laminin immunostaining in hyperplastic, dysplastic, and neoplastic lesions of the endometrium and uterine cervix Obstetrics & Gynecology 1987 69 5 794 799 2437505

[36] Bulletti C Galassi A Jasonni VM Martinelli G Tabanelli S Basement membrane components in normal hyperplastic and neoplastic endometrium Cancer 1988 62 1 142 149 10.1002/1097-0142(19880701)62:1<142::aid-cncr2820620124>3.0.co;2-y 3383111

[37] Barsky SH Siegal GP Jannotta F Liotta LA Loss of basement membrane components by invasive tumors but not by their benign counterparts Laboratory Investigation 1983 49 2 140 147 6348406

[38] Barsky SH Togo S Garbisa S Liotta LA Type IV collagenase immunoreactivity in invasive breast carcinoma Lancet 1983 1 8319 296 297 10.1016/s0140-6736(83)91708-7 6130313

[39] Laird SM Dalton CF Okon MA Bunning RA Marshall R Metalloproteinases and tissue inhibitor of metalloproteinase 1 (TIMP-1) in endometrial flushings from pre- and post-menopausal women and from women with endometrial adenocarcinoma Journal of Reproduction & Infertility 1999 115 2 225 232 10.1530/jrf.0.1150225 10434927

[40] Jeziorska M Nagase H Salamonsen LA Woolley DE Immunolocalization of the matrix metalloproteinases gelatinase B and stromelysin 1 in human endometrium throughout the menstrual cycle Journal of Reproduction & Infertility 1996 107 1 43 51 10.1530/jrf.0.1070043 8699433

[41] Rodgers WH Matrisian LM Giudice LC Dsupin B Cannon P Patterns of matrix metalloproteinase expression in cycling endometrium imply differential functions and regulation by steroid hormones Journal of Clinical Investigation 1994 94 3 946 953 10.1172/JCI117461 8083380 PMC295134

[42] Amalinei C Cianga C Balan R Cianga P Giusca S Immunohistochemical analysis of steroid receptors, proliferation markers, apoptosis related molecules, and gelatinases in non-neoplastic and neoplastic endometrium Annals of Anatomy 2011 193 1 43 55 10.1016/j.aanat.2010.09.009 21145716

[43] Iurlaro M Loverro G Vacca A Cormio G Ribatti D Angiogenesis extent and expression of matrix metalloproteinase-2 and -9 correlate with upgrading and myometrial invasion in endometrial carcinoma European Journal of Clinical Investigation 1999 29 9 793 801 10.1046/j.1365-2362.1999.00532.x 10469168

[44] Yu F Jiang Q Zhou Y Yang Z Yu X Abnormal expression of matrix metalloproteinase-9 (MMP9) correlates with clinical course in Chinese patients with endometrial cancer Disease Markers 2012 32 5 321 327 10.3233/DMA-2011-0886 22674412 PMC3826421

[45] Graesslin O Cortez A Uzan C Birembaut P Quereux C Endometrial tumor invasiveness is related to metalloproteinase 2 and tissue inhibitor of metalloproteinase 2 expressions International Journal of Gynecological Cancer 2006 16 5 1911 1917 10.1111/j.1525-1438.2006.00717.x 17009991

[46] Guo W Chen G Zhu C Wang H Expression of matrix metalloproteinase-2, 9 and it’s tissue inhibitor-1, 2 in endometrial carcinoma Zhonghua Fu Chan Ke Za Zhi 2002 37 10 604 607 10.3760/j.issn:0529-567X.2002.10.009 12487935

[47] Maity G Sen T Chatterjee A Laminin induces matrix metalloproteinase-9 expression and activation in human cervical cancer cell line (SiHa) Journal of Cancer Research and Clinical Oncology 2011 137 2 347 357 10.1007/s00432-010-0892-x 20425121 PMC11827979

[48] Koblinski JE Ahram M Sloane BF Unraveling the role of proteases in cancer Clinica Chimica Acta 2000 291 2 113 135 10.1016/s0009-8981(99)00224-7 10675719

[49] Ioachim E Charchanti A Briasoulis E Karavasilis V Tsanou H Immunohistochemical expression of extracellular matrix components tenascin, fibronectin, collagen type IV and laminin in breast cancer: their prognostic value and role in tumour invasion and progression European Journal of Cancer 2002 38 18 2362 2370 10.1016/s0959-8049(02)00210-1 12460779

[50] Zheng WQ Looi LM Cheah PL Correlation between laminin and cathepsin D expressions in breast carcinoma Tumori 2002 88 4 296 299 10.1177/030089160208800411 12400981

[51] Kikkawa Y Hozumi K Katagiri F Nomizu M Kleinman HK Laminin-111-derived peptides and cancer Cell Adhesion & Migration 2013 7 1 150 159 10.4161/cam.22827 23263633 PMC3544779

